# Stenting of the artery of Dr A.N. Kazantsev in the acute period of ischemic stroke

**DOI:** 10.1016/j.radcr.2022.07.034

**Published:** 2022-08-01

**Authors:** Anton N. Kazantsev, Alina S. Zharova, Ekaterina V. Sokolova, Alexander V. Korotkikh

**Affiliations:** a Kostroma regional clinical hospital named after E.I. Korolev, Kostroma, Russian Federation; b North-Western State Medical University. I.I. Mechnikov, St. Petersburg, Russian Federation; c Clinic of Cardiac Surgery of the Amur State Medical Academy of the Ministry of Health of Russia, Blagoveshchensk, Russian Federation

**Keywords:** Carotid angioplasty with stenting, Persistent embryonic hypoglossal artery, Carotid endarterectomy, Acute period of stroke, Stenting of the internal carotid artery, A.N. Kazantsev artery

## Abstract

The A.N. Kazantsev artery is a vessel starting from the common carotid artery with subsequent bifurcation into 2 vessels of equal size—the internal carotid artery (ICA) and the persistent embryonic hypoglossal artery (PEHA). Until now, this artery has been considered as the ICA. However, according to all existing classifications, the ICA in the cervical segment does not have arterial branches. In addition, in view of the comparable sizes of PEHA and ICA, PEHA itself cannot be considered a branch of the ICA. Thus, by the right of the first description, the authors of the article named this vascular formation as the A.N. Kazantsev artery, which forms a bifurcation of the PEHA and ICA. In this clinical case, carotid angioplasty (CAS) was performed with stenting of 80% stenosis of the A.N. Kazantsev artery in the most acute period of acute cerebrovascular accident (ACV). According to angiography, the following was also revealed: the presence of PEHA, extending from the A.N. Kazantsev artery 5 cm above its mouth, connecting with the main artery; stenosis of the right vertebral artery 60% at the mouth; hypoplastic left vertebral artery with aplasia of the V4 segment; open circle of Willis (VC): absence of both posterior communicating arteries (PCA). Due to the high risk of recurrent CVA due to clamping of the A.N. Kazantsev artery during CEA, a multidisciplinary consultation decided to implement an emergency CAS of the A.N. Kazantsev artery. The distal embolism protection system FilterWire was inserted into the proximal part of the basilar artery through the radial artery on the left. The distal embolism protection system RX Accunet was inserted into the distal parts of the left ICA through the left common femoral artery. According to Seldinger, an Acculink stent 7-10 × 30 mm was inserted into the affected area of the A.N. Kazantsev artery, positioned and opened. The postoperative period was uneventful. ACV did not recur. Conducted dual antiplatelet therapy (acetylsalicylic acid 125 mg in the afternoon + clopidogrel 75 mg in the morning). The patient was discharged from the institution on the 10th day after the operation in a satisfactory condition.

## Introduction

Emergency correction of hemodynamically significant stenosis of the internal carotid artery (ICA) is currently an important and little-studied section of carotid surgery [1], [2], [3], [4], [5]. Recent studies do not come to a consensus on the need for urgent interventions in the brachiocephalic system in the acute period of acute cerebrovascular accident (ACV) [6], [7], [8], [9], [10]. But the current Russian recommendations do not prohibit performing carotid endarterectomy (CEE) or (CAS) on an emergency basis [11]. Moreover, according to this document, the operation of choice is CEA, and CAS takes only an alternative position [11]. However, well-known domestic works are most often devoted to the analysis of the results of only one of these reconstruction methods on an emergency basis [12], [13], [14], [15], [16]. However, the only Russian multicenter study published in 2021 compared the results of both methods of revascularization [17]. The authors concluded that CEA is a less preferable option for correcting hemodynamically significant ICA stenosis in the urgent mode due to the high risk of developing hemorrhagic transformation of the ischemic focus in the brain and all adverse cardiovascular events [17]. Thus, according to the main conclusion of this study, ICA stenting is a safer procedure relative to open surgical interventions in patients in the most acute period of stroke [17]. Some difficulties are caused by the choice of strategy for emergency cerebral revascularization in the presence of a variant structure of the carotid bifurcation. In this situation, we are talking primarily about the persistent embryonic hypoglossal artery (PEHA), which connects the ICA and the basilar artery [18]. The frequency of its diagnosis in the general population does not exceed 0.3%, and with concomitant atherosclerosis of extracranial arteries, it is a rare exception [18]. To date, none of the major studies of the leading vascular centers in Russia have reported CEE or CAS in the presence of PEHA, which creates uncertainty in the choice of treatment strategy for this complex cohort of patients [19], [20], [21], [22], [23].

A separate issue concerns the terminology of the topographic anatomy of the carotid bifurcation under conditions of a functioning PEHA. Due to the rarity of this variant of the structure, in all atlases of human anatomy, and in particular, in the most popular publication in Russia, edited by Sinelnikov R.D., the following classification of parts of the ICA is distinguished: cervical, cavernous, stony and cerebral [24]. The cervical segment is understood as an area of the ICA that is localized extracranially and does not give off any arterial branches [24]. But since PEHA is precisely a branch of the ICA, then the definition of the term "cervical part of the ICA" loses its relevance. At its core, this segment will start from the place of origin of the PEHA to the entrance to the carotid canal, because it is in this area that the ICA has no other arterial branches. And the segment between the mouth of the ICA to the PEHA remains outside the nomenclature [24]. And if a hemodynamically significant stenosis is localized precisely in this zone, then it will be deprived of any exact name [24]. Thus, due to the lack of regulatory terminology, the authors of this work, who described an emergency reconstructive intervention on the arterial segment from the mouth of the ICA to the PEHA, introduced a new term "A.N. Kazantsev artery", which defines this segment of the carotid basin.

The purpose of this article was to describe a clinical case of emergency CAS of the A.N. Kazantsev artery in the most acute period of stroke.

## Clinical example

Man, 60 years old. He was admitted on an emergency basis to the State Budgetary Institution of Health “City Alexandrovskaya Hospital,” St. Petersburg. From the anamnesis, it is known that about an hour ago he felt weakness in the upper and lower extremities on the right, confusion of speech. By the time of admission to the institution, the neurological deficit had regressed. According to the data of multislice computed tomography of the brain, data for ischemic stroke were not established.

A color duplex scan of the brachiocephalic arteries was performed, according to which 80% stenosis of the left ICA was visualized with signs of unstable ASP (bleeding under the ASP cover). Five centimeter above the mouth of the ICA, there is an additional large branch extending into the cranium without signs of stenosis.

Examined by a neurologist, the diagnosis was made: transient ischemic attack in the basin of the left middle cerebral artery. A multidisciplinary consultation (neurologist, cardiovascular surgeon, endovascular surgeon, neurosurgeon, cardiologist, resuscitator, anesthesiologist) in view of the presence of hemodynamically significant stenosis of the left ICA with signs of unstable ASP, the absence of neurological deficit, decided to perform angiography with subsequent decision on the issue of emergency CAS.

**Fig. 1:**
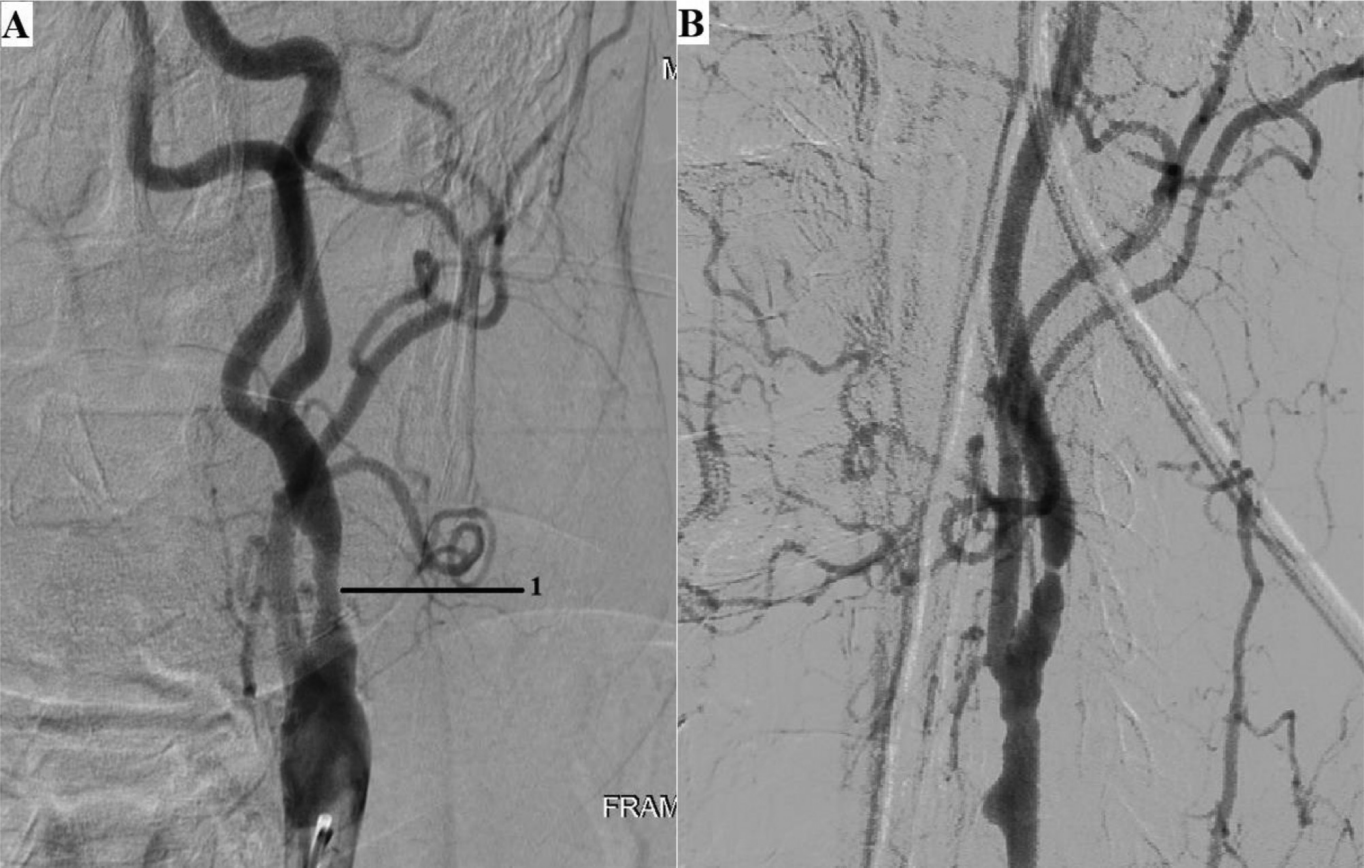
(A) Angiography of the carotid arteries on the left. 1—80% stenosis of the artery of A.N. Kazantsev (variant anatomy). (B) Angiography of the bifurcation of the left common carotid artery in another patient with normal anatomy (normal anatomy).

**Fig. 2:**
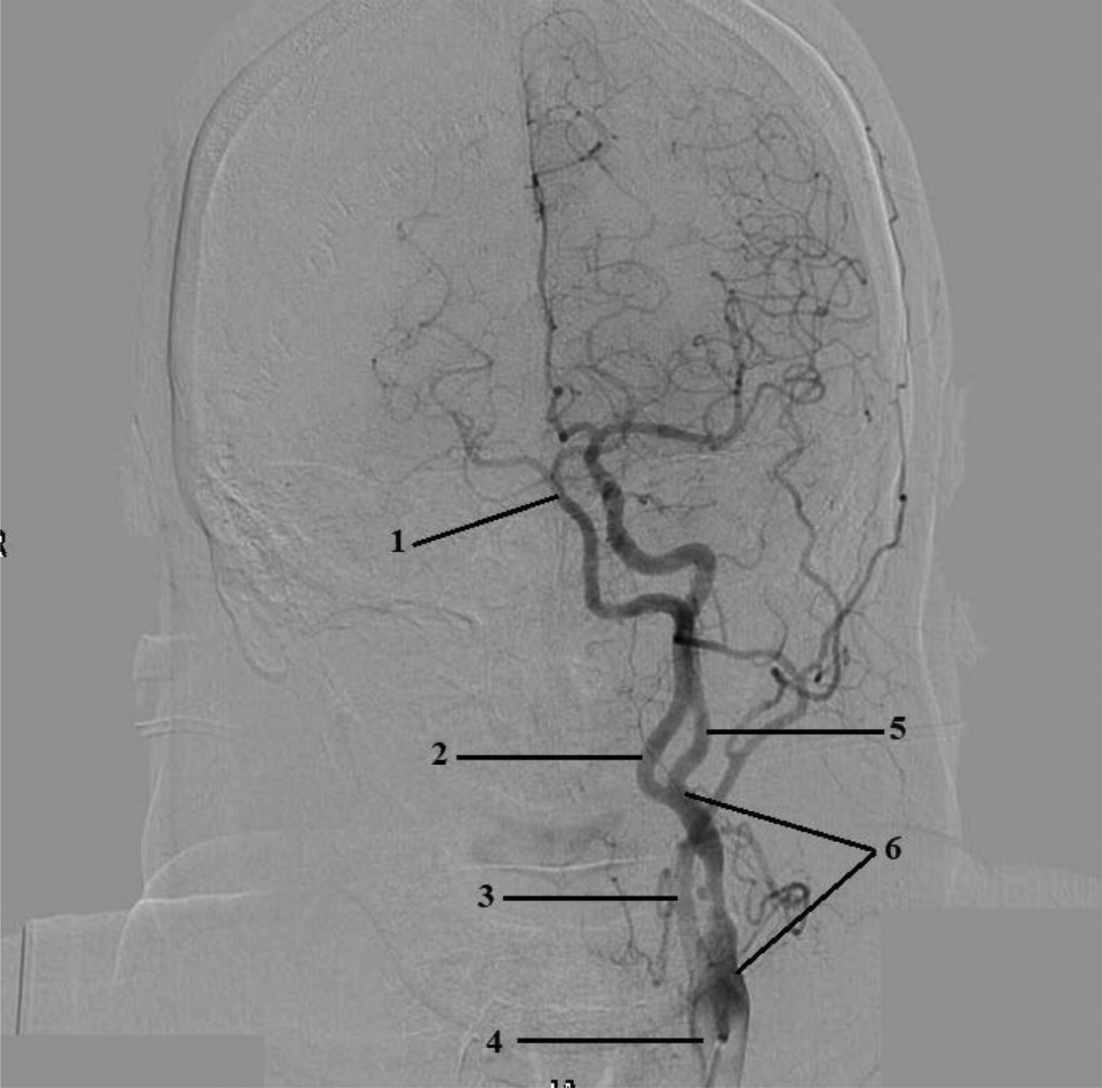
Angiography of the carotid arteries on the left and arteries of the Willis circle: 1—the main artery; 2—PEHA; 3—external carotid artery; 4—common carotid artery; 5—ICA; 6—A.N. Kazantsev artery.

**Fig. 3:**
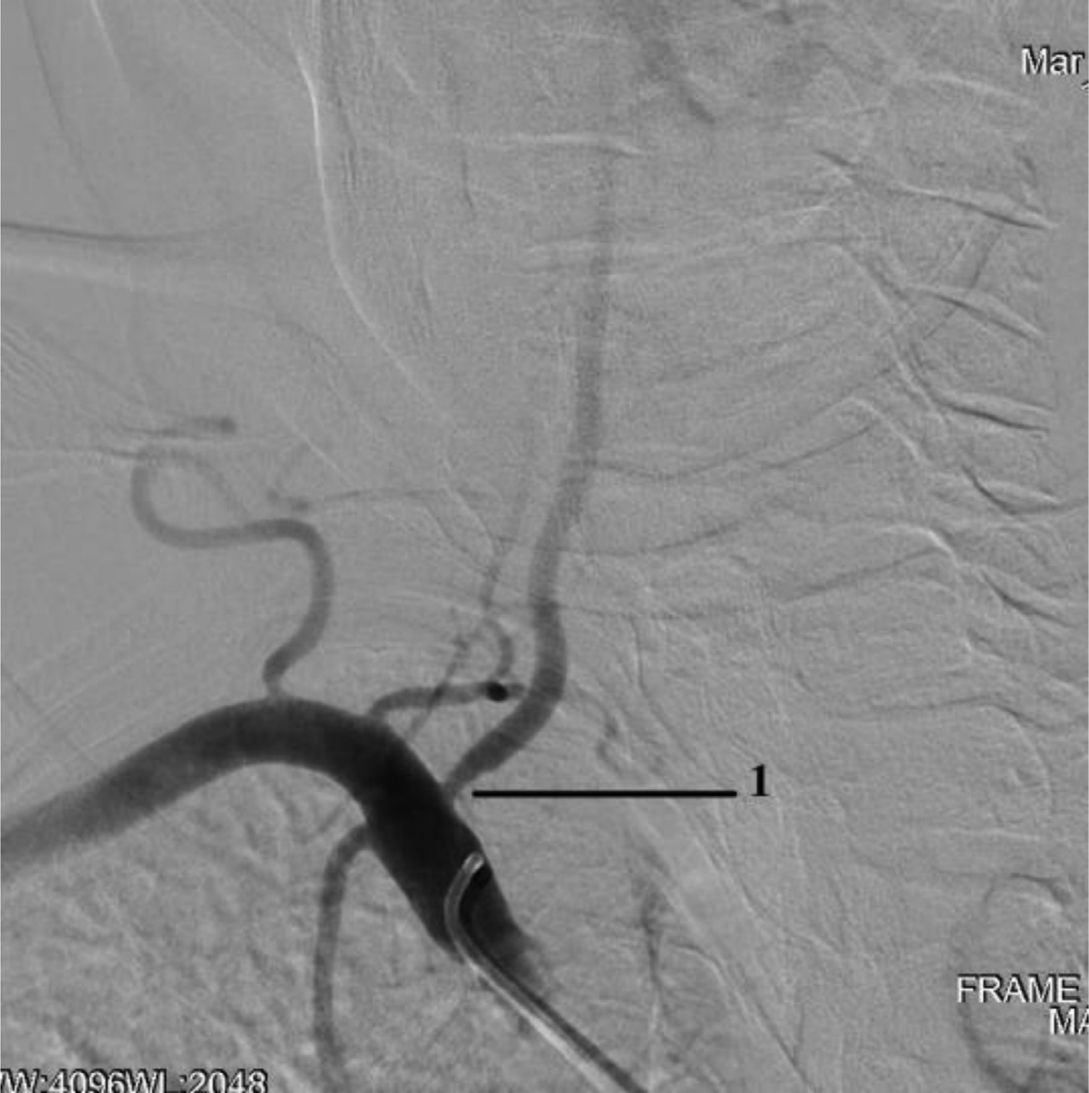
Angiography of the right subclavian and left vertebral arteries: 1—stenosis of the right vertebral artery 60% at the mouth.

**Fig. 4:**
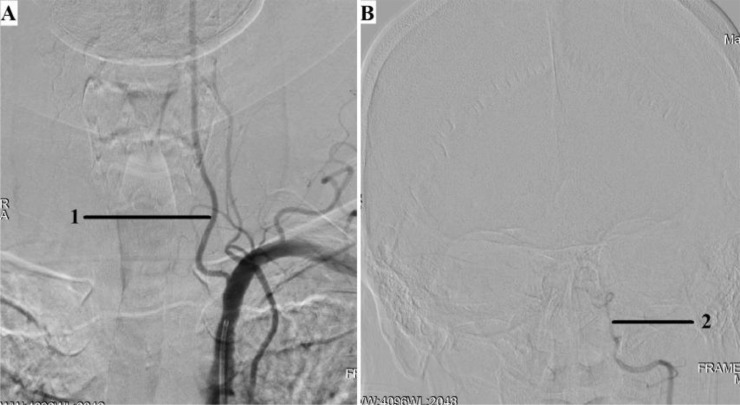
(A) Angiography of the left subclavian and left vertebral arteries; 1—hypoplasia of the left vertebral artery; (B) Angiography of the left vertebral artery; 2—aplasia of the V4 segment of the left vertebral artery.

**Fig. 5:**
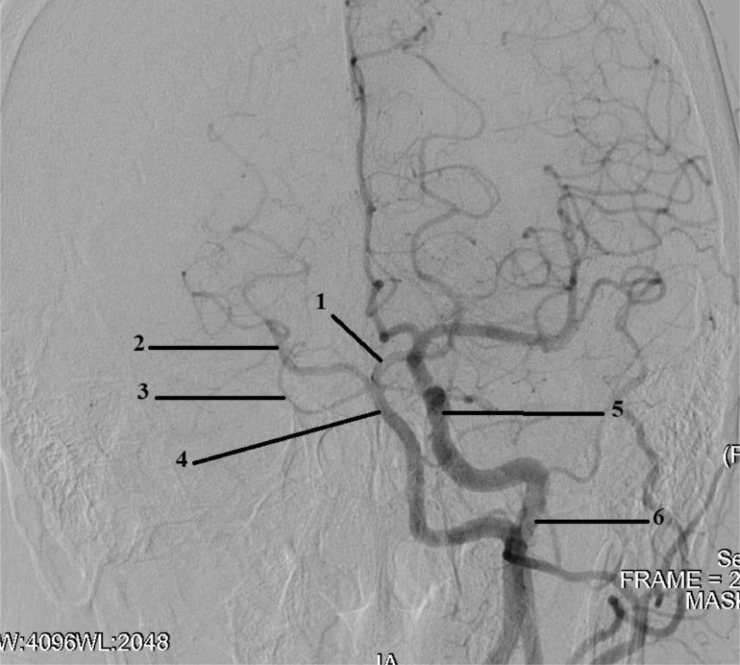
Angiography of the circle of Willis (open circle of Willis with the absence of both posterior connecting arteries): 1—left posterior cerebral artery; 2—the right posterior cerebral artery; 3—the right superior cerebellar artery; 4—the main artery; 5—middle cerebral artery; 6—internal carotid artery.

Angiography revealed: stenosis of the A.N. Kazantsev artery on the left 80% (Fig. 1), the presence of PEHA, extending from the A.N. Kazantsev artery 5 cm above its mouth, connecting with the main artery (Fig. 2); stenosis of the right vertebral artery 60% at the mouth (Fig. 3); hypoplastic left vertebral artery with aplasia of the V4 segment (Fig. 4); open circle of Willis

(VC): absence of both posterior communicating arteries (PCA) (Fig. 5).

Due to the high risk of recurrent CVA due to clamping of the A.N. Kazantsev artery during CEE, a multidisciplinary council (with the same composition) decided to implement an emergency CAS of the A.N. Kazantsev artery.

**Fig. 6:**
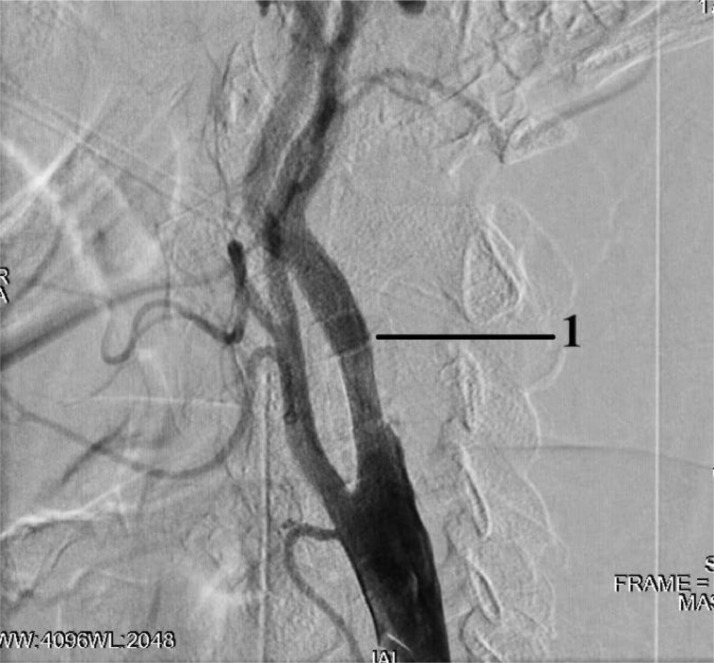
Stenting of the A.N. Kazantsev artery: 1—implanted stent.

The course of the operation: after processing the surgical field under m/a Sol. Lidocaini 0.2%—1 ml performed puncture of the right radial artery. Sheath 6F installed. A guiding catheter JR 5.0 6F SH was installed along the diagnostic guidewire at the mouth of the right vertebral artery. The distal embolism protection system FilterWire 3.5-5.5 mm was inserted into the proximal part of the basilar artery, and opened. On the diagnostic guidewire, the 6F sheath in the common femoral artery on the left is replaced by 7F. A JR 5.0 7F SH guiding catheter was inserted through the diagnostic guide into the left common carotid artery (CCA). The system of protection against distal embolism RX Accunet 6.5 mm was inserted into the distal parts of the left ICA. According to Seldinger, an Acculink stent 7-10 × 30 mm was inserted into the affected area of the A.N. Kazantsev artery, positioned and opened. The delivery system has been removed. On control angiography, residual stenosis in the area of stenting of the left ICA up to 60%, no signs of spasm. According to Seldinger, a balloon catheter Viatrac 5.5 × 30 mm was inserted into the area of residual stenosis. Performed postdilation at pressures up to 12 atm. The balloon catheter has been removed. Control angiography showed residual stenosis in the stenting area of the left ICA 0%, intracranial arteries without signs of embolism (Fig. 6).

The distal embolism protection systems were alternately removed from the ICA and the main arteries. The tool has been removed. Manual hemostasis of the puncture area of the right common femoral artery. Aseptic and pressure dressings. Manual hemostasis of the puncture area of the right radial artery. Aseptic and pressure bandages.

The postoperative period was uneventful. ACVE did not recur. Conducted dual antiplatelet therapy (acetylsalicylic acid 125 mg in the afternoon + clopidogrel 75 mg in the morning). The patient was discharged from the institution on the 10th day after the operation in a satisfactory condition.

## Discussion

The first and only report in Russia devoted to reconstructive intervention on the carotid arteries in the presence of PEHA was the article by Shanitsyn I.N. The authors achieved a successful result of revascularization with the implementation of CEE [18]. However, it should be noted that in the presence of PEHA, the vertebral arteries are significantly hypoplastic, and the PCA is most often aplastic [18]. An open VC and a deficit of collateral circulation in the vertebrobasilar basin during clamping of the ICA during CEA can lead to the development of ischemic stroke in this area of the brain. This condition most often manifests itself in edema and herniation of the trunk with the development of a lethal outcome [25]. The only option to maintain the constancy of hemodynamics during CEE is the installation of a temporary shunt (TS), which was described in the article by Shanitsyn I.N. But all “factory” samples of this device have only a 2-ended structure in order to start blood flow only from the CCA to the ICA [26], [27], [28].In the situation with PEHA during CEE, it is necessary to resort to improvisational solutions, either by creating a new 3-terminal version of the TS, or by using 2 TS separately from CCA to ICA and separately from CCA to PEHA [18]. It should be noted that these manipulations will be characterized by a high complexity of implementation, and “piling up” in the wound along with tourniquets and vascular clamps of the TS branches will significantly reduce the visualization of the reconstruction zone and make it difficult to perform anastomosis [18]. An additional aggravating factor is reports that the insertion of a VS in carotid surgery itself may be accompanied by distal embolism, dissection, and thrombosis of the ICA due to balloon inflation [26], [27], [28]. And if in the case of a typical structure, due to the consistency of the TS, it is most often possible to achieve a regression of the neurological deficit at the hospital stage of observation, then in the situation with PEHA and the absence of PCA, the same distal embolism can occur in the main artery, which will be accompanied by risk of fatal complications [18]. Thus, the totality of the facts presented indicates that CEE is the least preferred option for brain revascularization in patients with hemodynamically significant stenosis of the A.N. Kazantsev artery and a functioning PEHA.

In this article, we have demonstrated an effective way to treat this complex cohort of patients in CAS. An important manipulation that made it possible to achieve a successful outcome of the treatment was the use of 2 traps placed separately in the ICA and in the PEHA from different arterial accesses. This approach made it possible to maximally selectively prevent distal embolism during stent placement, which became a key moment in achieving the optimal result of revascularization.

It should also be noted that the choice in favor of emergency CAS and the rejection of CEA was made in view of the analysis of the latest Russian multicenter study that demonstrated a protective mechanism for the prevention of hemorrhagic transformation when using endovascular methods for correcting hemodynamically significant ICA stenosis in the urgent mode [17].

In addition, I would like to justify the author's name of the A.N. Kazantsev artery. Figure 1 shows that the diameter and dimensions of the PEHA are equivalent to the ICA. Thus, it is indisputable that PEHA is not correctly considered a branch of the ICA, it is an independent vessel. Based on this, the A.N. Kazantsev artery cannot be regarded as a segment of the ICA. It is also an independent vessel, the bifurcation of which forms PEHA and ICA. Thus, the A.N. Kazantsev artery must be considered an independent nomenclature unit of the brachiocephalic basin. Studies devoted to the results of studying the variant structures of the carotid bifurcation did not single out this arterial segment, considering it to be the ICA. However, based on the facts given above, it is impossible to consider the artery of A.N. Kazantsev ICA. It should also be noted that all known reports on the implementation of reconstructive interventions in such topographic conditions were carried out in a planned manner. And only in the last decade, surgeons have almost completely abandoned open operations (Table 1) on this arterial segment in favor of CAS (Table 2).

**Table 1:** Carotid endarterectomy in patients with persistent embryonic hyoid artery

First author	Year	Patient age	Gender	Operation side	Symptoms
Stern [29]	1978	57	Female	Right	Dizziness
Pinkerton [29]	1980	61	The male	Left	Stroke
Osgood [30]	1983	57	The male	Right	Amaurosis
Rodan [29]	1985	41	Female	left	TIA
Ouriel [29]	1988	Data not presented	Data not provided	Data not presented	TIA
McCartney [31]	1989	76	Female	Right	Amaurosis
Sunada [29]	1991	62	The male	Right	Stroke
Fantini [29]	1994	67	The male	Left	Stroke
Cartier [29]	1995	74	Female	Right	Is absent
Megyesi [31]	1997	72	The male	Right	TIA
Hatayama [29]	1999	71	Female	Left	Dizziness
Katoh [29]	1999	42	Female	Left	Fainting
Bertoletti [29]	2000	72	Female	Right	Is absent
Thayer [32]	2005	55	Female	Right	Is absent
Kawabori [33]	2009	71	The male	Right	Stroke

**Table 2:** Сarotid angioplasty with stenting with persistent embryonic hyoid artery

First author	Year	Patient age	Gender	Operation side	Symptoms
Kanazawa [29]	2008	68	Male	Left	обмороки
Nii [34]	2010	62	Male	Right	TIA
Silva [35]	2013	63	Feminine	Right	Stroke
Eller [36]	2013	60	Male	Right	Stroke
Huang [37]	2016	50	Feminine	Right	TIA
Muari [38]	2016	77	Male	Right	Stroke
Zhang [39]	2016	47	Male	Right	Is absent
Rya [40]	2016	60	Feminine	Left	Is absent

However, the authors did not describe the development of postoperative complications, demonstrating the efficacy and safety of both CAS and CEA.

It should be emphasized that in none of the above studies, the real artery was described as an independent formation. In addition, we analyzed 24 atlases of normal and pathological human topographic anatomy, in which this vascular formation was not distinguished into an independent artery either [41], [42], [43], [44], [45], [46], [47], [48], [49], [50], [51], [52], [53], [54], [55], [56], [57], [58], [59], [60], [61], [62], [63]. The authors described that the common carotid artery forms a bifurcation of the external carotid artery and the ICA, while the ICA goes to the skull without giving off branches [41], [42], [43], [44], [45], [46], [47], [48], [49], [50], [51], [52], [53], [54], [55], [56], [57], [58], [59], [60], [61], [62], [63]. The presence of PEHA has not been reported in these literature sources [41], [42], [43], [44], [45], [46], [47], [48], [49], [50], [51], [52], [53], [54], [55], [56], [57], [58], [59], [60], [61], [62], [63]. Thus, the segment of the artery corresponding to this characteristic corresponds to the vessel that starts from the bifurcation of the A.N. Kazantsev artery and then goes to the carotid canal. And the artery of A.N. Kazantsev, as a previously undescribed vessel, can bear the names of the authors who singled it out as a separate nomenclature unit.

This clinical case, according to the data of domestic (www.elibrary.ru) and foreign (www.pubmed.gov) electronic libraries, is the first report of a successful emergency CAS of the A.N. Kazantsev artery in the presence of a functioning PEHA in the most acute period of stroke, which demonstrates high relevance and can be used in the practice of vascular surgeons, neurosurgeons, endovascular surgeons, neurologists.

## Conclusion

CAS of hemodynamically significant stenosis of the A.N. Kazantsev artery is a safe and effective method of cerebral revascularization in the most acute period of stroke.

## Patient consent

The patient signed a voluntary consent to the use of his data and information about the treatment provided under this article.

## Appendix Supplementary materials

Image, application 1
