# Supplementary material for: Stenting of the artery of Dr A.N. Kazantsev in the acute period of ischemic stroke
Source: Radiol Case Rep. 2022 Aug 1;17(10):3699–708. doi: 10.1016/j.radcr.2022.07.034 (PMC9356102; doi:10.1016/j.radcr.2022.07.034)
Supplement: Supplementary file 1 [file mmc1.pdf]

ПРАВИТЕЛЬСТВО САНКТ-ПЕТЕРБУРГА

КОМИТЕТ ПО ЗДРАВООХРАНЕНИЮ

Санкт-Петербургское государственное бюджетное учреждение здравоохранения «Городская Александровская больница»

Пр. Солидарности, д. 4, г. Санкт-Петербург, 193312
Тел. (812) 583-16-23, факс (812) 589-11-41
ОКТМО 40386000 ОКОГУ 49003
ОГРН 1027806078255
ИНН/КПП 7811018700/781101001

_______________ № ____________________

на №_______________ от _________________

Editor-in-chief of the journal

The study was approved by the ethical committee of the Clinic of Cardiac Surgery of the Amur State Medical Academy of the Ministry of Health of Russia, Blagoveshchensk, Russian Federation. The patient signed consent to the publication of his data in this article. The study was performed in compliance with the ethical principles of scientific medical research involving humans. The work was performed in accordance with the standards of good clinical practice (Good Clinical Practice) and the principles of the Declaration of Helsinki, did not contradict the Federal Law of the Russian Federation of November 21, 2011 No. 323-FZ "On the basics of protecting the health of citizens in the Russian Federation", the order of the Ministry Health of the Russian Federation dated April 1, 2016 N 200n "On approval of the rules of good clinical practice".

The study has no funding.

Professor Kokov A.N.
